# Automatic freezing-tolerant rapeseed material recognition using UAV images and deep learning

**DOI:** 10.1186/s13007-022-00838-6

**Published:** 2022-01-13

**Authors:** Lili Li, Jiangwei Qiao, Jian Yao, Jie Li, Li Li

**Affiliations:** 1 School of Remote Sensing and Information Engineering, Wuhan University, Wuhan, China; 2 GongQing Institute of Science and Technology, Jiujiang, Jiangxi, China; 3 Key Laboratory of Biology and Genetic Improvement of Oil Crops, Ministry of Agriculture and Rural Affairs, Oil Crops Research Institute of the Chinese Academy of Agricultural Sciences, Wuhan, China; 4 School of Electrical and Electronic Engineering, Hubei University of Technology, Wuhan, China

**Keywords:** Freezing injury recognition, Rapeseed, UAV images, Deep learning, Machine learning

## Abstract

**Background:**

Freezing injury is a devastating yet common damage that occurs to winter rapeseed during the overwintering period which directly reduces the yield and causes heavy economic loss. Thus, it is an important and urgent task for crop breeders to find the freezing-tolerant rapeseed materials in the process of breeding. Existing large-scale freezing-tolerant rapeseed material recognition methods mainly rely on the field investigation conducted by the agricultural experts using some professional equipments. These methods are time-consuming, inefficient and laborious. In addition, the accuracy of these traditional methods depends heavily on the knowledge and experience of the experts.

**Methods:**

To solve these problems of existing methods, we propose a low-cost freezing-tolerant rapeseed material recognition approach using deep learning and unmanned aerial vehicle (UAV) images captured by a consumer UAV. We formulate the problem of freezing-tolerant material recognition as a binary classification problem, which can be solved well using deep learning. The proposed method can automatically and efficiently recognize the freezing-tolerant rapeseed materials from a large number of crop candidates. To train the deep learning network, we first manually construct the real dataset using the UAV images of rapeseed materials captured by the DJI Phantom 4 Pro V2.0. Then, five classic deep learning networks (AlexNet, VGGNet16, ResNet18, ResNet50 and GoogLeNet) are selected to perform the freezing-tolerant rapeseed material recognition.

**Result and conclusion:**

The accuracy of the five deep learning networks used in our work is all over 92%. Especially, ResNet50 provides the best accuracy (93.33$\%$) in this task. In addition, we also compare deep learning networks with traditional machine learning methods. The comparison results show that the deep learning-based methods significantly outperform the traditional machine learning-based methods in our task. The experimental results show that it is feasible to recognize the freezing-tolerant rapeseed using UAV images and deep learning.

## Background

As one of the most important economic crops in the world, rapeseed plays an essential role in ensuring the supply of edible vegetable oil and nutrient-rich feed. With the development of the global economy and the continuous improvement in the standard of living, the demand for rapeseed drastically increases. However, the supply of rapeseed in some countries is inadequate because of the limited arable land. For example, due to the serious shortage of arable land, China as the largest edible oil consumer in the world has to import large quantities of rapeseed oil to meet the increasing demand. In addition, farmers are reluctant to plant rapeseed in many countries as the price of fertilizer, pesticide and seed increases. The economic benefit of planting winter rapeseed decreases especially when the yield of rapeseed is not satisfactory. In order to meet the increasing demand for rapeseed, it is imperative to expand the planting area and increase the output per unit area of as much as possible.

There are many factors that influence the yield of rapeseed, such as drought, freezing injury and rainfall, etc. Among which, freezing injury is an important factor that affects the growth of rapeseed. Winter rapeseed often suffers from serious freezing injury during the overwintering period. Low temperature will directly inhibit the metabolic response and prevent the expression of the full genetic potential of the plant [1]. The nutrient transport through rapeseed stems is also affected by freezing injury, which leads to the decrease of seed setting rate and yield. Thus, it is necessary for breeders to breed freezing-tolerant rapeseed materials. By doing so, the rapeseed planting area in cold regions can be expanded. Rapeseed better adapted to cold climates can reduce the yield loss from freezing injury. However, in the past few years, the freezing-tolerant rapeseed material recognition still relies on field investigation conducted by agricultural experts. Experts need to select the freezing-tolerant materials from a huge number of crop candidates, which is time-consuming, error-prone, and inefficient. It is necessary to propose a new approach to recognize the freezing-tolerant materials automatically and efficiently. The development of the remote sensing technology makes it possible.

In the field of remote sensing, the satellite images are one of the most powerful and important data. Spectral information of the satellite images can express the relationship between natural environmental conditions and vegetation disasters. In recent years, satellite images have been widely applied to recognition of the crop damage caused by drought [2, 3], flood [4, 5], hail [6, 7] and freezing injury [8, 9]. Because the normalized difference vegetation index (NDVI) of crop changes dramatically after suffering freezing injury, the NDVI is a prominent feature to analyze the freezing injury. Some studies compared and analyzed the NDVI calculated by satellite images before and after freezing injury to recognize the crop freezing damage [10–13]. However, the plant area of each candidate material is usually small (about 2 square meters). The satellite data with a limited spatial resolution can not recognize each material accurately. Thus, it is difficult to use satellite images to select the freezing-tolerant materials. With the development of unmanned aerial vehicles (UAVs), the images captured by UAVs provide another new source data to recognize the freezing-tolerant materials.

Compared with satellite images, the images captured by the sensors mounted on UAVs have a higher spatial resolution. Multispectral or hyperspectral images captured by the UAVs are one of the widely used source images in the field of agriculture. Multispectral or hyperspectral images can provide abundant and accurate spectral information of crops. Recently, many researchers proposed to use multispectral or hyperspectral images collected by UAVs to recognize the crop freezing injury [14–16]. In general, different vegetation indices (VIs) are calculated using source images, and multiple VIs are combined to recognize the freezing-injured crops. However, because the acquisition and processing of multispectral or hyperspectral data are relatively complicated, it is hard for non-professional users to acquire high-quality multispectral images using the sensor mounted on an UAV platform. Nowadays, the consumer UAVs equipped with an RGB camera provide a new choice for recognizing freezing-tolerant materials. Compared with the professional UAV equipped with a multispectral sensor, the consumer UAV has the advantage of being cheap, convenient and flexible.

Consumer UAVs outfitted with an RGB camera have been widely used to monitor the crop growth process. With the advancement of artificial intelligence (AI), the combination of UAVs and AI has been widely applied to different tasks in agriculture, such as disease and insect detection [17–19], yield prediction [20, 21], and crop lodging detection [22, 23]. Recently, the combination of RGB images collected by UAVs and AI also has been applied to detect and classify the crop stresses [24–26]. Su et al. [27] developed a supervised learning system based on support vector machine (SVM) to recognize the crop water stress using RGB images collected by an UAV. Firouz et al. [28] applied SVM to recognize the healthy and freezing damaged citrus fruits. However, the performance of traditional machine learning models depends heavily on the hand-crafted features designed using the image information and prior knowledge. It is difficult to manually design the optimal features for different crop stress tasks. The traditional machine learning models are difficult to meet the requirements for crop stress recognition. With the rise of deep learning technologies, many deep learning-based solutions have been proposed for different crop stress recognition tasks.

Nowadays, deep learning has made great breakthroughs and has been widely used in crop stress recognition. The advantage of deep learning is that it provides powerful feature representations through learning, the feature of images will be learned automatically by deep learning models [29]. In recent years, some researchers proposed to recognize the crop stresses using deep learning instead of traditional machine learning [30, 31]. Yang et al. [32] adopted a convolutional neural network (CNN) model to extract spectral features in the visible-near-infrared range. They then used the extracted spectral features to estimate the maize seedling freezing damage. The freezing damage detected by CNN has a high correlation with the ranking given by chemical methods, which proves that spectral analysis based on the CNN model is suitable for recognizing the freezing damage in corn seedlings. Anami et al. [33] adopted a deep convolutional neural network (DCNN) framework to automatically recognize and classify various biotic and abiotic stresses of rice using the RGB images collected by an UAV. The accuracy of the recognition result is 92.89%, which proves the potential of deep learning in recognizing crop stress. The fact proves that deep learning is more suitable for recognizing crop stress than traditional machine learning. Deep learning provides an effective way to assist experts in selecting stress-tolerant materials.

In this paper, we proposed a low-cost freezing injury recognition method using UAV images and deep learning. The main purpose of this research is to develop an approach that combines RGB images captured by a consumer UAV with deep learning to automatically recognize the freezing-tolerant rapeseed materials from a large number of breeding candidates. The breeders can then quickly assess and select the breeding materials from the freezing-tolerant materials that are preliminarily selected by deep learning. Preliminary selection among a large number of crop materials candidates is an important step for breeders.

## Dataset and methodology

**Fig. 1:**
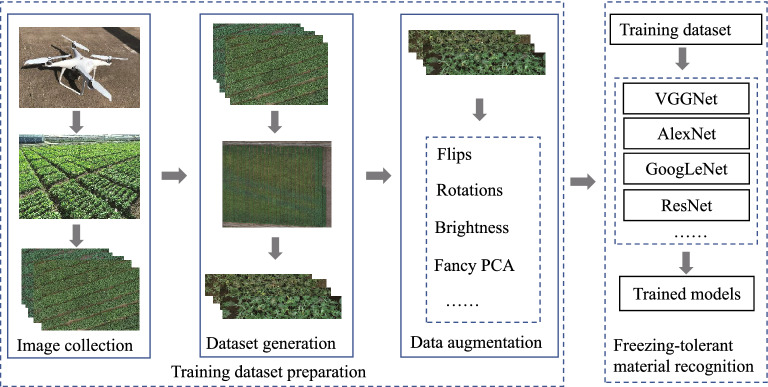
The workflow of our proposed deep learning-based freezing-tolerant rapeseed material recognition approach

To automatically and efficiently recognize the freezing-tolerant rapeseed materials from a large number of candidates, we propose a new deep learning-based freezing injury recognition approach using the RGB images collected by a consumer UAV. The whole workflow of the proposed approach is presented in Fig. 1.

### Overview of study area and data source

#### Study area

**Fig. 2:**
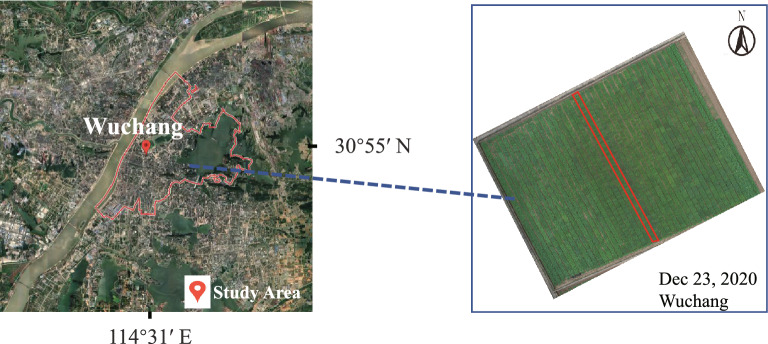
Overview of the test site used in our work

The experiment was conducted at the Oil Crops Research Institute, Chinese Academy of Agricultural Sciences Wuchang, Wuhan, Hubei province, China ($114^{\circ }$31′N, $30^{\circ }$55′E, with an elevation of 20 m). The test site location is shown in Fig. 2. The test site is divided into 42 plots covering an area of about 0.45 ha. The red box in Fig. 2 represents a plot, and the plots range in length from 56 to 61 m. The width of each plot is about 2 meters. Each plot consists of approximate 300 rows. The line spacing of two adjacent rows is about 16.7 cm. There are more than 3000 pure materials. Each rapeseed material consists of three rows. The rapeseed was sown on September 27th, 2020. To ensure that the growth of all materials before the overwintering period is normal, we carried out a continuous observation on rapeseed and took remedial measures for abnormal materials. The key goal of our proposed approach is to automatically and effectively select these freezing-tolerant materials from all materials using UAV images and deep learning.

#### Image acquisition

**Fig. 3:**
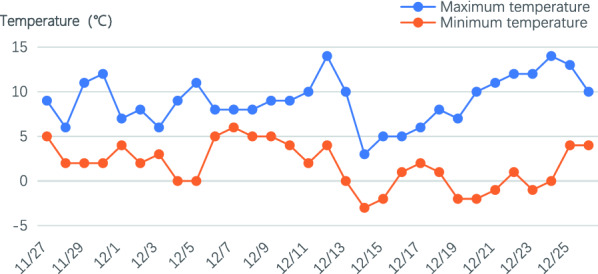
Temperature chart of Wuhan in November to December 2020

In Fig. 3, we present the part of historical temperature chart of Wuhan in November and December 2020. From the observation of temperature chart and field investigation, we find that the rapeseed materials have suffered from the damage of low temperature around December 20th, 2020. In our experiments, we collected the UAV images of this test site on December 23th, 2020. The images were acquired by a DJI Phantom 4 Pro V2.0 (DJI, Shenzhen, China) equipped with an RGB camera with a spatial resolution of $5472 \times 3648$ pixels. The flight campaign was conducted from 3:00 pm to 3:30 pm. The weather is cloudy without wind. The flight height and speed are about 10 m and 1.8 m/s, respectively. In order to generate the orthophoto map successfully, we set the frontal and side overlap as 75% and 75%, respectively.

#### Dataset generation

In this paper, we formulate the freezing-tolerant material recognition as a classification problem, which can be solved well using deep learning. However, deep learning is a data-driven method, we need to prepare a large number of samples with ground truth to train the model. In this study, we directly apply the Agisoft PhotoScan software (Agisoft LLC, St. Petersburg, Russia) to generate the orthophoto map of the test site. Agisoft PhotoScan is an excellent software, which is applied to automatically generate the high-resolution real orthophoto map and the DEM model. The orthophoto image is then used to create the rapeseed freezing injury recognition dataset.

After the orthophoto map generation, the Adobe Photoshop software is used to cut out images to generate the experimental dataset. In the practical application of large-scale rapeseed fields, we can crop images in batches based on the geographic coordinates of the rapeseed fields, so as to generate large numbers of cropped images automatically and efficiently. As mentioned above, every material consists of three rows. Thus, we cut out the orthophoto map into 2847 samples, each sample represents a material. All samples are resized to $600 \times 150$ pixels after cropping. Three experts evaluated the rapeseed freezing injury according to the freezing injury phenotype in the field. After error elimination, the evaluation results were then taken as the final labels of experimental data. There are about three symptoms of rapeseed freezing injury, including the freezing injury of weak seedlings, leaves, and bolting. The symptoms of freezing injury to weak seedlings are that the roots are lifted and uprooted, and rapeseed will die when temperatures rise and the soil thaws. The symptoms of freezing injury to leaves are that the leaves are yellow or fuchsia and shrivelled, and the petioles of some rapeseed materials are water-soaked. As temperatures rise and the soil thaws, the leaves gradually wilt, turn yellow, and fall off. The symptoms of freezing injury to the bolting are mainly manifested as the cuticle of the stalk breaking, the bolting stalks being badly broken off, and the rapeseed dying when temperatures rise. The labels of freezing-injured samples and freezing-tolerant samples are 1 and 0, respectively. Then, we divide the whole dataset into the training dataset and the test dataset with an 8:2 ratio randomly. As a result, 2277 images are used to train the deep learning network and 570 images are used to test the accuracy of the trained model. The detailed numbers of samples in the training and the test datasets are shown in Table 1.

**Table 1:** The number of freezing-injured and freezing-tolerant samples in the training and test datasets

Dataset	Freezing-tolerant	Freezing-injured
Training dataset (80%)	572	1705
Test dataset (20%)	94	476

**Fig. 4:**
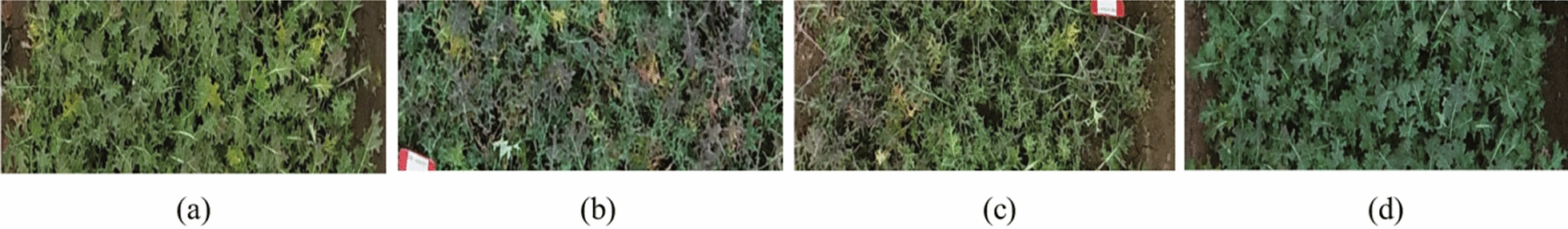
The symptoms of the freezing-injured and the freezing-tolerant materials. The freezing-injured material with yellowish leaves (**a**), dark, fuchsia leaves (**b**), overturned leaves and water-soaked petioles. **c**, **d** are the freezing-tolerant materials with normal leaf colors and petioles

The obvious rapeseed freezing injury symptoms are shown in Fig. 4. Wilson [34] pointed out that the symptoms of freezing-injured plants can be seen on leaves, including leaf wilting, leaf bleaching, or in extreme cases, plant death. The freezing-injured material has the following symptoms: the leaves turn yellow (see Fig. 4a), the leaf colors is dark or fuchsia (see Fig. 4b), the leave is overturned and the petiole has wet blotch (see Fig. 4c). The leaf colors and petioles of the freezing-tolerant materials are normal, as shown in Fig. 4d.

#### Dataset augmentation

**Fig. 5:**
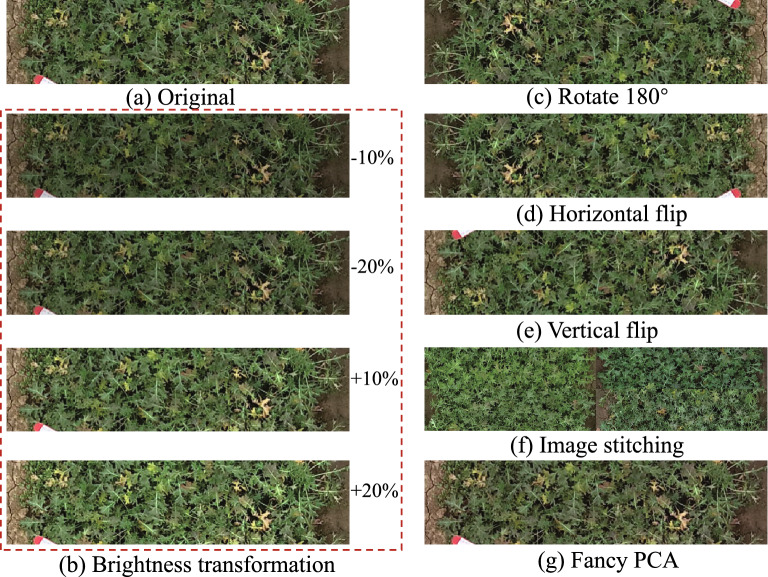
The example results of data augmentation strategies used in our study: **a** original image, **b** brightness transformation, **c**–**e** geometric transformation, **f** image stitching strategy and (**g**) fancy PCA

Deep learning is a data-driven approach, the number and quality of the training dataset will directly influence the effect of the trained model. In general, as the number of high-quality samples increases, the performance of the deep learning-based approach increases. However, in real applications, the number of high-quality samples is often insufficient. To get a high-quality trained model and improve its generalization ability, we need to increase the number of the dataset. Geometric transformation, brightness transformation, image stitching and fancy PCA (Principal Components Analysis) are used in our study for data augmentation. The results of these strategies are shown in Fig. 5.

To make the model more robust to the brightness changes, we use the brightness transformation which is a common data augmentation strategy. We first transform the images from RGB to HSV color space. Then, we adjust the brightness of the original image. The original image brightness is adjusted to 80%, 90%, 110% and 120%, respectively. The example result of brightness transformation is shown in Fig. 5b.

The geometric transformation is also a common image augmentation strategy, including rotation, flip operation, etc. The rotation operation and flip operation are used to augment the dataset in this study. The results of geometric transformation are shown in Fig. 5c–e, respectively.

As shown in Fig. 4, there are many symptoms of freezing-injured rapeseed. In the real environment, the situation of freezing injury is more complex. In order to improve the generalization ability of the model for case of freezing injury, we adopt the diagonal stitching strategy. We randomly select four images with the same label in the dataset and stitch the four images into one new image. Then, we resize the stitching image to the original image size of $600 \times 150$ pixels. The example result of diagonal stitching strategy is shown in Fig. 5f.

Fancy PCA as a common data augmentation strategy is firstly proposed in the study of AlexNet [35]. Fancy PCA changes the intensities of the RGB channels along with the natural variation of the images and performs PCA on the color channels. Firstly, PCA is performed on all RGB pixel values to obtain the eigenvectors ($\hbox {P}_1$, $\hbox {P}_2$, $\hbox {P}_3$) and eigenvalues ($\lambda$ $_1$, $\lambda$ $_2$, $\lambda$ $_3$), and then a set of random values ($\alpha$ $_1$, $\alpha$ $_2$, $\alpha$ $_3$) from a Gaussian distribution with mean = 0 and standard deviation = 0.1 times eigenvalues to get the [$\alpha$ $_1$ $\lambda$ $_1$, $\alpha$ $_2$ $\lambda$ $_2$, $\alpha$ $_3$ $\lambda$ $_3$]. Finally, to each RGB image pixel $Ixy=[I_{xy}^{R}, I_{xy}^{G}, I_{xy}^{B}]$, the quantity $[$ $\hbox {P}_1$, $\hbox {P}_2$, $\hbox {P}_3$ $][$ $\alpha$ $_1$ $\lambda$ $_1$, $\alpha$ $_2$ $\lambda$ $_2$, $\alpha$ $_3$ $\lambda$ $_3$ $]^{\mathsf {T}}$ is added. The description detail of the Fancy PCA is reported in [35]. Fancy PCA is used to augment the dataset in this study, the example result of fancy PCA is shown in Fig. 5g.

### Architectures of CNNs

**Fig. 6:**
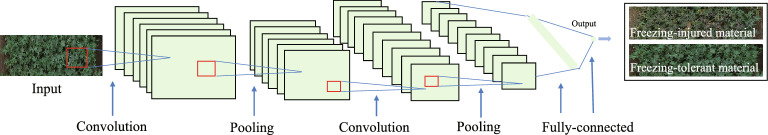
Architecture of the classical CNN

To effectively distinguish between freezing-injured and freezing-tolerant materials, we propose to apply a convolutional neural network (CNN) to classify the images of all materials. In Fig. 6, we present a basic CNN architecture designed for the classification of rapeseed materials. In general, a CNN architecture consists of a set of convolutional, pooling and fully connected layers. In our paper, we directly select several classical CNN architectures to complete the task for freezing-tolerant rapeseed material recognition.

#### AlexNet

AlexNet [35] is a popular CNN architecture that was the first to win the ImageNet contest. In addition, AlexNet is the first network to implement a deep convolutional neural network structure on a large-scale image dataset. AlexNet architecture consists of 5 convolutional layers, 3 max-pooling layers, 2 normalization layers, 2 fully connected layers, and 1 softmax layer. Instead of using the sigmoid or tanh activation functions, AlexNet uses the Rectified Linear Unit (ReLU) activation function. Krizhevsky et al. [35] found out that using ReLU as an activation function can significantly accelerate the speed of the training process. AlexNet additionally employs dropout layers in order to avoid overfitting.

#### VGGNet

VGGNet [36], which came in the second place in the 2014 ImageNet Challenge, explores the relation between the depth of convolutional neural networks and the model performance. VGGNet has 11–19 levels of depth, with VGGNet16 and VGGNet19 being the common networks. The VGGNet16 is used in this study. VGGNet16 has 13 convolutional layers, 5 max-pooling layers, 2 normalization layers, 3 fully connected layers, and 1 softmax layer. The improvement of VGGNet is that consecutive $3 \times 3$ convolution kernels are used to replace the larger convolution kernels ($11 \times 11$, $7 \times 7$ and $5 \times 5$). The main purpose of consecutive $3 \times 3$ convolution kernels is to improve the effectiveness of the neural network by increasing the depth of the network while maintaining the same perceptual field. The VGGNet proves that the representation depth is beneficial to the classification accuracy.

#### GoogLeNet

Increasing the network depth is an effective method to improve the performance of the deep neural network. However, as the number of network layers increases, more parameters and processing resources would be required. As the champion of ILSVRC14, GoogLeNet [37] proposed the Inception structure which improves the utilization of the network computing resources. The Inception module is assembled in parallel by multiple convolutions and pooling operations. The GoogLeNet network consists of the Inception modules stacked upon each other. The detailed introduction of the Inception module is reported in [37]. In this study, we use a common GoogLeNet architecture that consists of multiple Inception modules, which assembled in parallel by $1 \times 1$, $3 \times 3$ and $5 \times 5$ convolutional filters followed a $3 \times 3$ max pooling.

#### ResNet

The depth of the neural network is important for the model performance. However, the degradation problem occurs when the deeper network starts converging. As one of the breakthroughs in the field of computer vision in recent years, ResNet [38] proposes a deep residual learning framework using shortcut connections to solve the degradation problem. The shortcut connection is an identity mapping that enables information to flow across layers without attenuation caused by nonlinear transformations of multiple stacks [39]. Two types of residual building blocks are proposed for different depth networks to reduce the number of parameters. For deeper networks, it consists of $1 \times 1$, $3 \times 3$ and $1 \times 1$ convolutions with a shortcut connection. For shallow networks, it consists of $3 \times 3$ and $3 \times 3$ convolutions with a shortcut connection. The depths of ResNet range from 18, 34, 50, 101 to 152 layers. The ResNet18 and ResNet50 are used in our study to recognize the rapeseed freezing injury.

## Experiment and analysis

In this section, to verify the effectiveness of the proposed method, we conducted the following experiments. To prove the validity of the method and illustrate the performance of different networks, we trained the five CNN models and tested the performance of different models. To prove the performance of CNN models is better than the traditional machine learning models, we compared the performance of four traditional machine learning models and the five CNN models. To test the effectiveness of the data augmentation strategy for CNN models, we compared the performance of the five models before and after data augmentation strategy. In addition, we used the new test site image collected by a consumer UAV to test the generalization ability of different models. All CNN model architectures are implemented in Python using the PyTorch on Ubuntu operating system. The memory of the processor is 128G, and the GPU is NVIDIA GTX 1080Ti. All traditional machine learning models are implemented in Python on Windows 10 operating system. It has a GPU of NVIDIA force RTX 3070, an Intel^®^$\hbox {Core}^{\mathrm{TM}}$ i7-10700 CPU @ 3.20 GHz with 128 GB RAM.

### Evaluation metrics

The task of this study is to distinguish between freezing-injured and freezing-tolerant materials from a large number of candidates. The freezing injury recognition is a binary classification problem. To evaluate the performance of models used in the study, we use the common binary classification evaluation metrics, including accuracy, precision, recall and F-score. The four evaluation metrics are defined as:

1 $$\begin{aligned} \left\{ \begin{aligned}&accuracy=\frac{TP+TN}{TP+TN+FP+FN} ,\\&precision=\frac{TP}{TP+ FP} ,\\&recall=\frac{TP}{TP+ FN} ,\\&F\text {-}score=2\times \frac{precidion\times recall}{precidion + recall}, \end{aligned} \right. \end{aligned}$$

where *TP* and *TN* denote the numbers that are correctly predicted as freezing-tolerant and freezing-injured materials, respectively. *FN* denotes the number of freezing-tolerant materials that is incorrectly predicted as freezing-injured materials. *FP* denotes the number of freezing-injured materials that is incorrectly predicted as freezing-tolerant materials.

The accuracy is the most intuitive evaluation metric for the overall performance of the model. However, it may fail when the number of samples from different categories is unbalanced. Thus, we further use the recall, precision and F-score to compare the performance of models. The precision measures the percentage of the correctly predicted freezing-tolerant materials over all predicted freezing-tolerant materials in the dataset. The recall measures the percentage of the correctly predicted freezing-tolerant materials over all the freezing-tolerant materials in the dataset. The F-score is the harmonic mean of precision and recall.

### Comparison of different CNNs

In this section, five common CNN models (AlexNet, VGGNet16, ResNet18, ResNet50 and GoogLeNet) were used to classify the freezing-injured and the freezing-tolerant materials. In all experiments, we used the cross-entropy loss function and the SGD optimizer with a learning rate of 0.01, and the batch size of each model is 16.

Table 2 shows the comparison results of classification performance of different CNN models. Firstly, it can be seen that the accuracy of the five models is all over 92%, which indicates that CNN algorithms show promise in the freezing injury recognition of rapeseed. Next, we can see that the ResNet50 achieves the highest accuracy (93.33%) among the five models. However, the accuracy can be affected by unbalanced samples, it cannot fully reflect the performance of the CNN models. For example, in this study, the category we are interested in is freezing-tolerant materials. However, when the number of two categories is unbalanced that the number of freezing-tolerant materials is far less than the freezing-injured materials, freezing-tolerant materials that are misclassified as freezing-injured materials can still make the model achieve high accuracy. Thus, we further use the recall, precision and F-score to evaluate the performance of CNN models.

The model with a high recall (low FN) means that the model can correctly identify a large number of freezing-tolerant materials. The value of recall will be considered if the breeders want to find the maximum number of possible freezing-tolerant materials. The model with high precision (low FP) means that a small number of freezing-injured materials are recognized as freezing-tolerant materials. Breeders can quickly pick out the small amounts of freezing-injured materials from the results generated by deep networks. If we only consider the labor cost savings of selecting freezing-tolerant materials, ResNet18 will be recommended due to its highest precision (85.53%). Because the aim of breeding is to select as many freezing-tolerant materials as possible. If only recall and precision are considered as evaluation metrics, recall is a more important evaluation metric for the recognition of the materials. Thus, ResNet50 will be recommended because of the highest recall (79.79%) while achieving the highest accuracy of 93.33%.

In the real situation, the breeders hope that they can quickly select more freezing-tolerant materials from candidates, the values of precision and recall should all be high. However, precision and recall are often conflicting. Hence, we need to balance two metrics. F-score is a weighted average of precision and recall. If the difference between the values of FP and FN is large, then the F-score should be considered firstly [40]. Among the five CNN models, ResNet50 obtains the highest F-score of 79.79%. Overall, ResNet50 outperforms other CNN models for the freezing-tolerant rapeseed material recognition.

From the above analysis, we can conclude that it is feasible to recognize the freezing-tolerant rapeseed material using UAV images and deep learning. In addition, the ResNet50 performs best among all selected networks. The ResNet50 offers the best scores of accuracy (93.33%), recall (79.79%) and F-score (79.79%).

**Table 2:** Quantitative evaluation results of five CNNs

Models	Accuracy (%)	Recall (%)	Precision (%)	F-score (%)
AlexNet	92.63	73.40	80.23	76.67
VGGNet16	93.16	73.40	83.13	77.97
GoogLeNet	92.45	67.02	84.00	74.56
ResNet18	92.98	69.15	**85**.**53**	76.47
ResNet50	**93**.**33**	**79**.**79**	79.79	**79**.**79**

### Comparison of traditional machine learning models and CNN models

In this section, we compared the performance of four traditional machine learning models and five CNN models. In the traditional machine learning models, the SIFT [41] and SURF [42] are selected as the hand-crafted features. SIFT and SURF are the two most widely used features and have been applied in many fields of agriculture, such as crop disease [40, 43], crop/weed classification [44], etc. In addition, the SVM and artificial neural network (ANN) are selected as the classifier.

The comparison results of four traditional machine learning models and five CNN models are shown in Table 3. As can be observed from the table, the evaluation metrics of the five CNN models are greater than those of the traditional machine learning models. The CNN networks significantly outperform the traditional machine learning models in our task because of the strong feature representation ability. To support real-time processing, computation time is also an important component to consider [40]. The single image testing times of the nine models are shown in Table 3. The CNN models take longer than traditional machine learning models for computation time of a single image due to the model complexity. The ResNet50 has the longest single image testing time compared to all other algorithms. Compared with AlexNet, which takes the least time, ResNet50 takes nearly 5 times to test a single image. However, in practical application, the time of 47 ms is much less than manual selection.

**Table 3:** Comparison results between traditional machine learning and CNNs

Type	Models	Accuracy (%)	Recall (%)	Precision (%)	F-score (%)	Testing times (ms)
Traditional machine learning	SIFT+SVM	67.72	30.85	19.59	**23**.**97**	2.66
	SURF+SVM	61.05	**36**.**17**	17.35	23.45	**0**.**59**
	SIFT+ANN	**76**.**32**	15.96	**21**.**43**	18.2 9	7.84
	SURF+ANN	72.98	12.77	18.46	15.09	4.41
CNNs	AlexNet	92.63	73.40	80.23	76.67	**10**.**16**
	VGGNet16	93.16	73.40	83.13	77.97	10.88
	GoogLeNet	92.45	67.02	84.00	74.56	22.32
	ResNet18	92.98	69.15	**85**.**53**	76.47	36.54
	ResNet50	**93**.**33**	**79**.**79**	79.79	**79**.**79**	47.25

### Illustration of data augmentation strategies

In this section, we compared the performances of five CNN models before and after data augmentation. We augmented the dataset (2847 images) following the data augmentation strategy described above. The number of final dataset was expanded by 10 times, reaching 28470 images in total. The comparison results of the five CNN models before and after data augmentation are shown in Table 4. From the table, we can see that the accuracy of five models is improved using the data augmentation strategy. However, the maximum improvement of the model accuracy is 0.53%, which is relatively small. In addition, the other three evaluation metrics (recall, precision and F-score) do not improve significantly after using the data augmentation, and some of the models even show a decrease in the scores of evaluation metrics. It is shown that the data augmentation methods mentioned above have little effect on the accuracy of the freezing-tolerant rapeseed material recognition. The reason is that the boundary of the division between rapeseed freezing-injured and freezing-tolerant samples is not clear enough. The increase of fuzzy data will affect the performance of the CNN models. We also observed that the ResNet50 outperforms the other CNN models after the data augmentation. It is consistent with the observation presented above.

**Table 4:** Comparison results before and after data augmentation of five CNNs

Dataset	Models	Accurac y (%)	Recall (%)	Precision (%)	F-score (%)
Original dataset	AlexNet	92.63	73.40	80.23	76.67
	VGGNet16	93.16	73.40	83.13	77.97
	GoogLeNet	92.45	67.02	84.00	74.56
	ResNet18	92.98	69.15	**85**.**53**	76.47
	ResNet50	**93**.**33**	**79**.**79**	79.79	**79**.**79**
Augmented dataset	AlexNet	92.98	69.15	85.53	76.47
	VGGNet16	93.68	76.60	83.72	80.00
	GoogLeNet	92.80	71.28	82.72	76.57
	ResNet18	93.33	70.21	**86**.**84**	77.65
	ResNet50	**93**.**68**	**79**.**79**	81.52	**80**.**65**

### Experiment on the new test site (Wuchang) in 2020

**Fig. 7:**
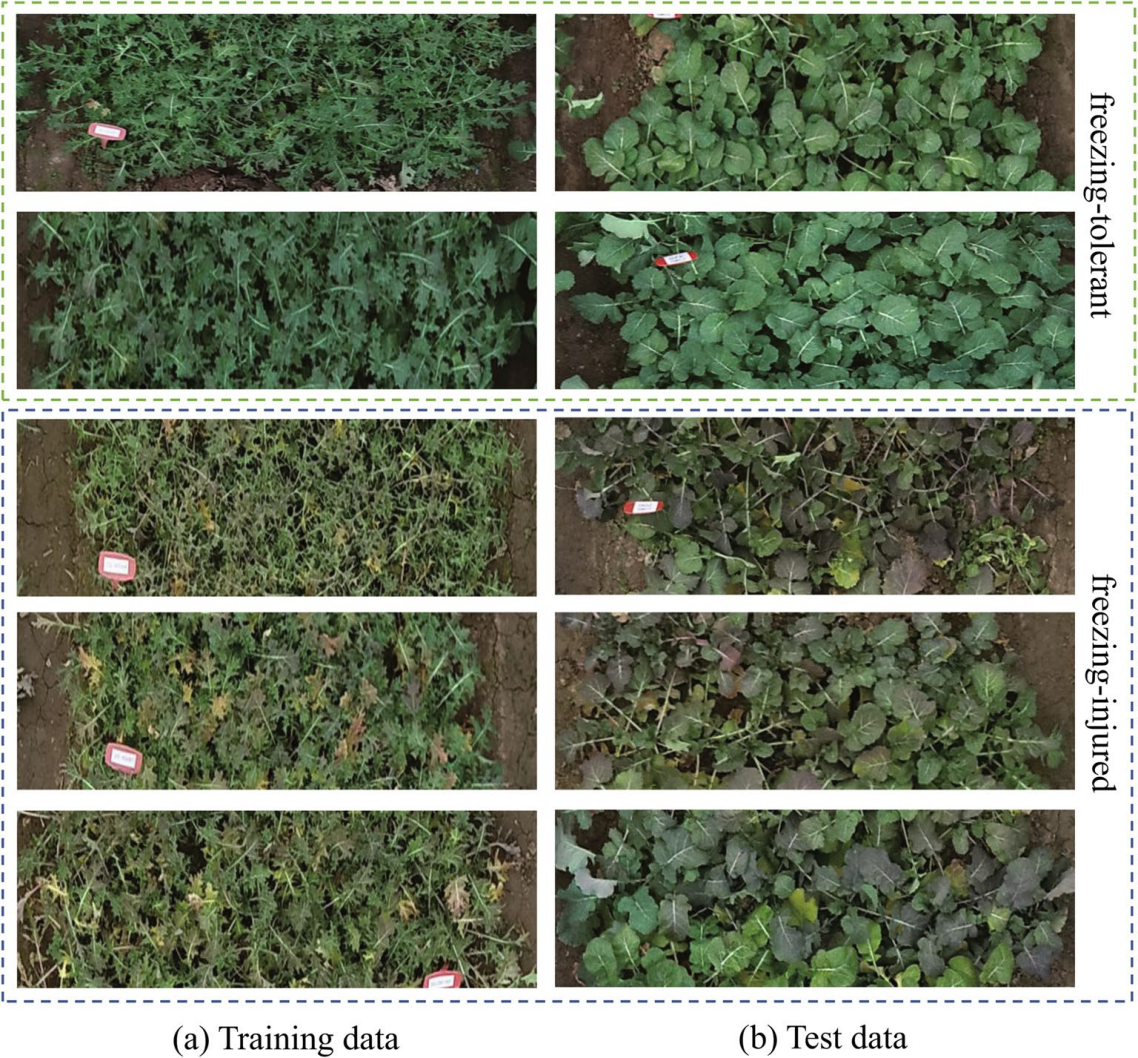
Comparision of sample data between training dataset (**a**) and test dataset (**b**)

**Fig. 8:**
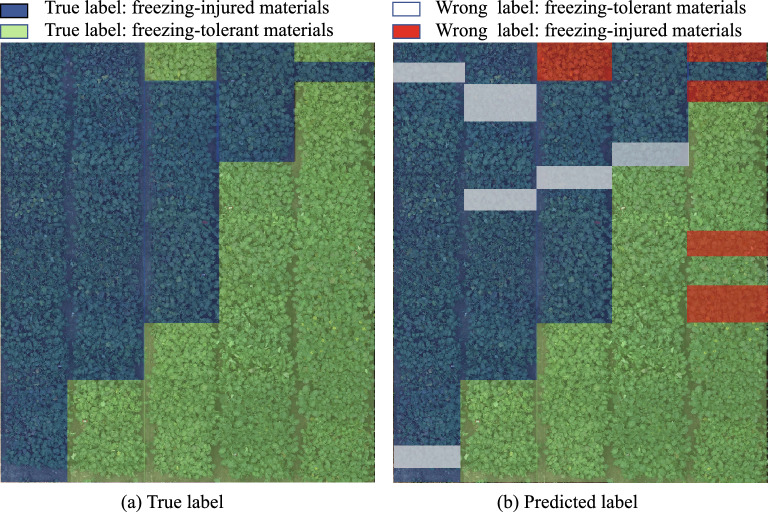
Visualization for recognition result of ResNet50 at new test site of Wuchang

In this section, we used the images collected from a new site (Wuchang) to test the generalization ability of the trained models. Generalization ability refers to the ability of a trained model that can make accurate predictions for the new data. We selected 175 materials including 80 freezing-tolerant materials and 95 freezing-injured materials from the new test site. In Fig. 7, we presented several images selected from the training dataset and the new test site. There is a large difference in the leaf shape between the training and test images in the percentage of leaf area and the degree of leaf loss. The different leaf shapes are designed to make the light transmittance of rapeseed leaves different. Part of the rapeseed materials at the new test site are shown in Fig. 8a. The green boxes represent the freezing-tolerant materials and the blue boxes represent the freezing-injured materials.

In the above experiments, the ResNet50 outperforms other CNN models, SIFT+ANN has the highest accuracy and SIFT+SVM has the highest F-score among the four traditional machine learning methods. Thus, we used the ResNet50, SIFT+ANN and SIFT+SVM to test the images collected from the new test site. The comparison results are shown in Table 5. Although there is a large difference in the training dataset and the test dataset, the recognition performance of the CNN models outperforms traditional machine learning models on the new dataset. The generalization ability of the ResNet50 can basically meet the requirement of freezing-tolerant rapeseed material recognition.

The visualization result of ResNet50 is shown in Fig. 8b. The red boxes and white boxes represent materials that are recognized incorrectly by the ResNet50. By visualizing the experimental result of the model, we can visually observe that the freezing injury of rapeseed in large fields. Breeders can quickly assess and select freezing-tolerant materials based on the visualization result. In addition, breeders can take remedial action for early freezing-injured materials and reduce the loss of rapeseed freezing injury. The visualization of the rapeseed field can help managers for accurate management in complex and large-scale fields.

**Table 5:** Experimental results of three models at the new test site (Wuchang). (T-FT and T-FI denote that the true labels of rapeseed materials are freezing-tolerant and freezing-injured, respectively. P-FT and P-FI denote that the predicted labels of rapeseed materials are freezing-tolerant and freezing-injured, respectively)

Models		T-FT	T-FI	Accuracy (%)	Recall (%)	Precision (%)	F-score (%)
ResNet50	P-FT	63	17	**85**.**71**	**88**.**73**	**78**.**75**	**83**.**44**
	P-FI	8	87				
SIFT+ANN	P-FT	63	17	46.29	45.00	78.75	57.27
	P-FI	77	18				
SIFT+SVM	P-FT	37	43	55.43	46.25	51.39	48.68
	P-FI	35	60				

### Experiment on the new test site (Xinzhou) in 2021

**Fig. 9:**
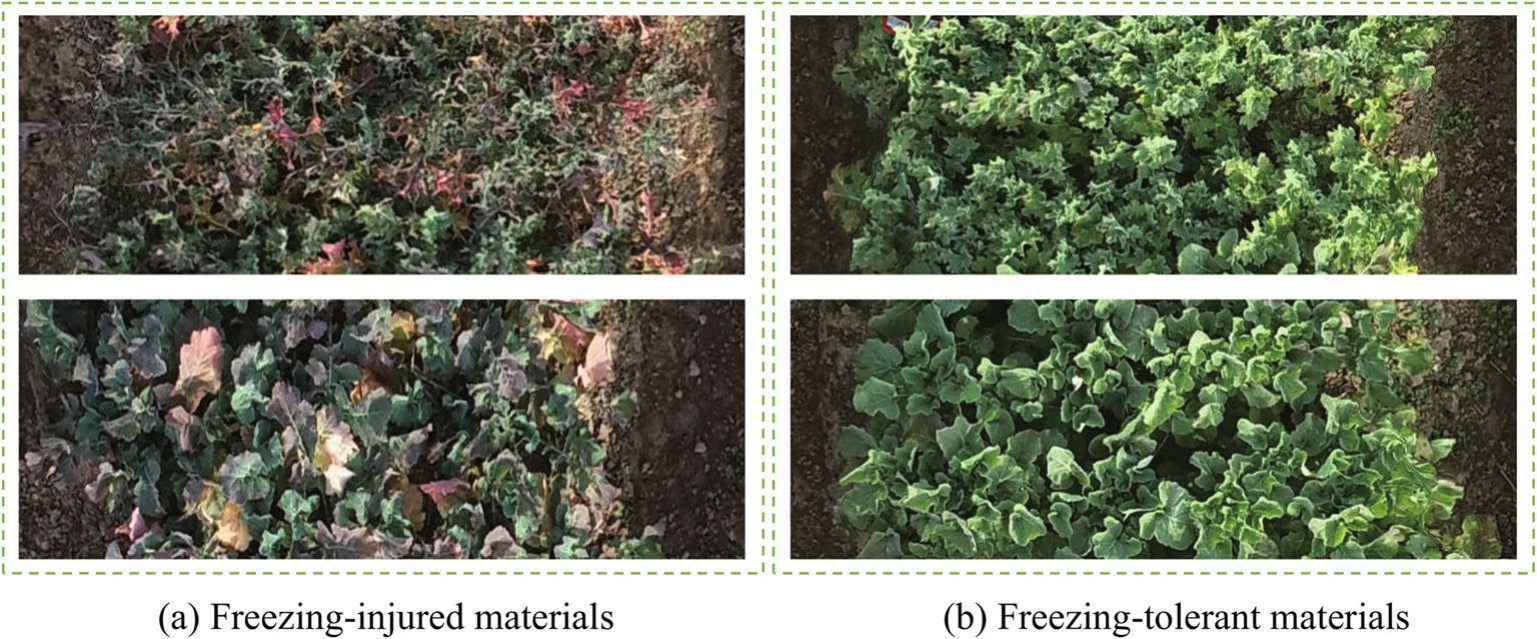
Comparision of sample data between the freezing-injured (**a**) and the freezing-tolerant materials (**b**) at Xinzhou

To further verify the generalization performance of the model in other regions, we collected the rapeseed images at the Oil Crops Research Institute, Chinese Academy of Agricultural Sciences, Xinzhou, Wuhan, Hubei province, China. The planting environment at this test site is consistent with that in Wuchang. The rapeseed was sown on September 27th, 2021. We collected the UAV images of this test site on December 7th, 2021. We selected 204 materials including 91 freezing-tolerant materials and 113 freezing-injured materials from the test site. The materials we selected included two kinds of leaf-shaped materials. The part of the test image is shown in Fig. 9.

Table 6 shows the test result of ResNet50. Overall, it achieves the accuracy of 84.80%. The recall, precision and F-score are 81.25%, 85.71% and 83.42%, respectively. Next, we will train the model using more overwintering rapeseed images collected in multiple regions and on multiple dates to further improve the recognition performance of the model.

**Table 6:** Experimental results of ResNet50 at Xinzhou. (T-FT and T-FI denote that the true labels of rapeseed materials are freezing-tolerant and freezing-injured, respectively. P-FT and P-FI denote that the predicted labels of rapeseed materials are freezing-tolerant and freezing-injured, respectively)

Model		T-FT	T-FI	Accuracy (%)	Recall (%)	Precision (%)	F-score (%)
ResNet50	P-FT	78	13	**84**.**80**	**81**.**25**	**85**.**71**	**83**.**42**
	P-FI	18	95				

## Conclusion

This study combines deep learning and UAV images to build an AI-assisted freezing-tolerant rapeseed material recognition model. This method can recognize freezing-tolerant materials from a large number of materials and further assist breeders in selecting breeding materials. In this study, the accuracy of the five CNN models was all over 92%. In addition, ResNet50 achieves the highest accuracy of 93.33%, while achieving the highest F-score of 79.79% and the highest recall of 79.79% among the other models. It proves the feasibility of freezing-tolerant rapeseed material recognition using UAV images and deep learning.

The freezing-tolerant rapeseed material recognition method established in this study has the advantages of economy, convenience, automation, and high precision. Breeders can not only quickly assess and select freezing-tolerant materials but also take remedial action for early freezing-injured materials to reduce the losses. This method can provide strong support for the scientific research work of rapeseed breeding and the study of the mechanism of freezing tolerance in rapeseed. Visualization of the recognition result will further help breeders to achieve accurate management of rapeseed freezing injury.

Although some fine results have been obtained, there are still some improvements that may be taken into consideration in future work. We will collect rapeseed images during the overwintering period on multiple dates and in multiple regions to increase the diversity of data. In addition, we will divide the materials into more refined categories according to the freezing resistance of the materials to further help the breeders select the required materials more efficiently. We will also apply this method to other adversity monitoring and evaluation of rapeseed to promote accurate and intelligent development in the rapeseed field.

## Data Availability

Not applicable.
